# Perceptual Correlates of Turkish Word Stress and Their Contribution to Automatic Lexical Access: Evidence from Early ERP Components

**DOI:** 10.3389/fnins.2016.00007

**Published:** 2016-01-20

**Authors:** Hatice Zora, Mattias Heldner, Iris-Corinna Schwarz

**Affiliations:** Department of Linguistics, Stockholm UniversityStockholm, Sweden

**Keywords:** Turkish, prosody, word stress, lexical access, event-related potential

## Abstract

Perceptual correlates of Turkish word stress and their contribution to lexical access were studied using the mismatch negativity (MMN) component in event-related potentials (ERPs). The MMN was expected to indicate if segmentally identical Turkish words were distinguished on the sole basis of prosodic features such as fundamental frequency (*f*_0_), spectral emphasis (SE), and duration. The salience of these features in lexical access was expected to be reflected in the amplitude of MMN responses. In a multi-deviant oddball paradigm, neural responses to changes in *f*_0_, SE, and duration individually, as well as to all three features combined, were recorded for words and pseudowords presented to 14 native speakers of Turkish. The word and pseudoword contrast was used to differentiate language-related effects from acoustic-change effects on the neural responses. First and in line with previous findings, the overall MMN was maximal over frontal and central scalp locations. Second, changes in prosodic features elicited neural responses both in words and pseudowords, confirming the brain's automatic response to any change in auditory input. However, there were processing differences between the prosodic features, most significantly in *f*_0_: While *f*_0_ manipulation elicited a slightly right-lateralized frontally-maximal MMN in words, it elicited a frontal P3a in pseudowords. Considering that P3a is associated with involuntary allocation of attention to salient changes, the manipulations of *f*_0_ in the absence of lexical processing lead to an intentional evaluation of pitch change. *f*_0_ is therefore claimed to be lexically specified in Turkish. Rather than combined features, individual prosodic features differentiate language-related effects from acoustic-change effects. The present study confirms that segmentally identical words can be distinguished on the basis of prosodic information alone, and establishes the salience of *f*_0_ in lexical access.

## Introduction

Turkish stress assignment has so far mostly been investigated from a phonological point of view, with default word stress being reported to be ordinarily on the final syllable (Lees, 1961; Sezer, 1983; Hameed, 1985; Barker, 1989; Inkelas and Orgun, 1998, 2003; Kabak and Vogel, 2001). This default pattern is argued to be neither sensitive to morphological nor to rhythmical considerations; word stress (henceforth stress) is always final regardless of the suffixes attached to the stem as in *taní* “know,” *tan̓-dík*, “acquaintance,” *tan̓d̓k-lár* “acquaintances” (Sezer, 1983). However, a number of exceptions have been reported, and one of them is associated with irregular roots. In contrast to regular roots that are stressed on the final syllable, irregular roots (e.g., place names) follow a quantity-sensitive rule (Sezer, 1983). According to this rule, stress placement is determined by the syllabic structure: Stress occurs on the antepenult if the penult is light and antepenult is heavy as in Á*nkara*; otherwise it falls on the penult as in Í*stánbul*. Regular and irregular roots occasionally create minimal pairs, which are segmentally identical but different in prosodic features such as *bebék* “baby” and *bébek* “a district in Ístanbul.” Given that prosodic features play a crucial role in lexical access (Cutler et al., 1997; Friedrich et al., 2004; Zora et al., 2015), even in languages where the number of word pairs distinguished by prosodic features alone is small (Cutler, 2005), this study investigates the impact of prosodic features in the activation of such segmentally identical Turkish pairs without any given context.

The opinions on the properties of Turkish stress have diverged widely in the literature (for an overview of Turkish stress in the first half of 20th century, see Excursus on Stress in Lees, 1961). Although most of the work on this topic has not made a distinction between stress- and pitch-accent to refer to the prominent syllable of a word, some authors have introduced this distinction and claimed Turkish to be a pitch-accent language rather than a stress-accent language^1^ (Underhill, 1986; Levi, 2005). Other authors have claimed that there is a difference in the manifestation of final and non-final stress in Turkish; while stress is realized by a pitch-accent on the final syllable, it is realized by a stress-accent on the non-final syllable (Csató and Johanson, 1998; Johanson, 1998).

There are very few studies investigating the phonetic aspects of Turkish stress (Konrot, 1981; Levi, 2005; Pycha, 2006), and these studies have focused on the acoustic correlates rather than on the perceptual ones. These studies have investigated the production of Turkish stress by looking at another exception, which is associated with pre-stressing suffixes. In contrast to stressable suffixes, pre-stressing suffixes require that the stress falls on the preceding adjacent syllable. Konrot (1981), for instance, investigated the role of fundamental frequency (*f*_0_), intensity, duration, and vowel quality in disyllabic minimal pairs created by stressable noun-making suffix—mA^2^ and pre-stressing negative suffix—mA (*kazmá* “pickaxe” vs. *kázma* “do not dig”). Findings indicated only *f*_0_ and intensity as correlates, and furthermore that the correlates were only used in non-final positions. Similarly, Levi (2005) investigated the role of *f*_0_, intensity and duration in minimal pairs created by stressable locative suffix—dA and pre-stressing instrumental suffix—lA (*metindé* “in the text” vs. *metínle* “with the text”) and by stressable infinitive suffix—mAk and pre-stressing negative suffix—mA (*gezmék* “to travel” vs. *gézmemek* “not to travel”). Findings indicated that the stress was realized differently in final and non-final positions. A discriminant analysis confirmed that *f*_0_ was the most robust correlate to mark stress, followed by intensity and then by duration. However, duration and intensity were argued to be less reliable than *f*_0_; furthermore, it was claimed that duration is unlikely to be used in the perception of stressed syllables. Pycha (2006) examined acoustic correlates by comparing simplex nouns having final stress and their suffixed versions created by pre-stressing interrogative suffix—mI (*dedé* “grandfather” vs. *dedé mi* “grandfather?”). Here, stress had multiple correlates in both final and non-final positions. However, *f*_0_ was argued to be the primary correlate of stress in both positions despite the non-robustness of *f*_0_ in the final position. The differences in *f*_0_, intensity, duration, and vowel quality were all shown to be statistically significant. However, only *f*_0_ was argued to function as a perceptual cue because the average differences in intensity and duration between stressed and unstressed positions were below the perceptual threshold of just-noticeable differences.

The studies reviewed above investigated the acoustic manifestation of Turkish stress by comparing final and non-final stress in complex words created by stressable and pre-stressing suffixes. The findings established *f*_0_ as the most salient cue in marking stressed syllables. However, there was disagreement as to where and to what extent prosodic features are employed: While some identified both *f*_0_ and intensity as correlates, but only in non-final position (Konrot, 1981), others identified *f*_0_, intensity and duration as correlates for both final and non-final positions, but argued that only *f*_0_ can function as perceptual correlate (Pycha, 2006). It should be noted that these studies employed complex words and therefore failed to avoid morphological effects. Moreover, they were not independent from sentence-level prosody; since both pre-stressing negative suffix—mA and pre-stressing interrogative suffix—mI form a full sentence, they may introduce an imperative and a question contour, respectively.

To date, only one study of stress perception in Turkish has employed the ERP technique (Domahs et al., 2013). This study investigated the sensitivity to violations of predictable and unpredictable stress patterns. Monomorphemic trisyllabic words with three different stress patterns such as in *fiasco* /fiyásko/, ^*^/fíyasko/, and ^*^/fiyaskó/ were examined. In line with the acoustic findings above, the results indicated that final stress was processed differently from non-final stress. Different stress violations elicited different ERP components: While stress violations with final stress elicited an N400 component, violations with non-final stress produced a P300 component. It was argued that the application of the predictable default (final) pattern to the words with non-final stress resulted in higher costs in lexical processing. In contrast, the application of the unpredictable pattern to the words with final-stress resulted in the evaluation of this pattern. It was, accordingly, argued that Turkish speakers were less responsive to stress shifts that lead to final stress patterns than to stress shifts that resulted in non-final stress patterns. The study evaluated this finding with regard to the typology of stress-deafness, which suggests that native speakers of languages with predictable stress are less sensitive to stress variations than native speakers of languages with variable stress (Peperkamp and Dupoux, 2002). Thus, stress-deafness in Turkish was claimed to occur only for the final stress pattern. This finding is in agreement with the above-mentioned acoustic studies that found a weakening of acoustic correlates for final stress. However, neither the ERP study nor the acoustic studies were concerned with establishing perceptual correlates of stress in Turkish and their role in lexical access.

So far, no empirical research using the ERP technique has addressed perceptual correlates of stress in Turkish. Perceptual correlates and their role in lexical access can be examined using the mismatch negativity (MMN) component. The MMN is a neurophysiological measure that signals the brain's automatic response not only to any acoustic change in the auditory sensory input (e.g., changes in *f*_0_, intensity and duration) but also to higher cognitive processes such as the activation of long-term memory traces for lexical information (Dehaene-Lambertz, 1997; Näätänen et al., 1997, 2007; Näätänen and Winkler, 1999; Winkler et al., 1999; Näätänen, 2001; Pulvermüller et al., 2001; Shtyrov and Pulvermüller, 2002; Zora et al., 2015). The MMN is elicited irrespective of the subject's attention to the auditory stimulus and is based on an oddball paradigm. That is, the MMN is elicited when a rare stimulus (deviant) is interspersed among frequent stimuli (standard; Näätänen et al., 1978; Näätänen and Winkler, 1999; for a review, see Näätänen et al., 2007).

In the present study, we studied the neural responses to changes in *f*_0_, spectral emphasis, and duration in Turkish words that differ in stress but are identical in segmental structure. Spectral emphasis was used rather than overall intensity to better reflect the role of loudness in stress perception. Spectral emphasis is characterized by the relative intensity in the higher frequency bands and has been shown to be a more reliable correlate than overall intensity in both production and perception (Sluijter and van Heuven, 1996; Sluijter et al., 1997; Heldner, 2003). A pseudoword pair with both possible stress patterns was used as control. The pseudoword pair was included to enable a comparison between ERP correlates of prosodic cues on the basis of lexical processing and non-lexical processing. The neural responses were recorded in relation to the second syllable of words and pseudowords in an auditory oddball paradigm by presenting four deviants interspersed among standard stimuli. The deviants differed from the standard in (i) *f*_0_, (ii) spectral emphasis, and (iii) duration alone as well as in (iv) all features combined. It was hypothesized that the MMN would indicate if segmentally identical Turkish words are distinguished on the basis of these features alone, and that their salience and relevance in lexical access would be reflected in the amplitude of MMN responses.

## Methods

### Participants

The participants were 14 native speakers of Turkish (10 males, four females; age range 20–54 years, *M* = 30.8, *SD* = 7.8), currently residing in Stockholm, Sweden. The general inclusion criteria were age of moving to Sweden (≥12 years, *M* = 24.7, *SD* = 7.2) and length of residence (≤ 15 years, *M* = 6.1, *SD* = 5.1). Handedness was assessed by the Edinburgh Handedness Inventory (Oldfield, 1971); all participants were right-handed. All of them reported normal development and hearing. The participants were rewarded with movie tickets for their participation. Informed consent was signed prior to testing, and the study was approved by the Stockholm Regional Ethics Committee (2015/63-31).

### Materials and manipulations

The experiment consisted of one word block and one pseudoword block. The material in the word block was a monomorphemic disyllabic Turkish minimal pair in which the location of stress on the first or second syllable led the word to be identified either as *a district in İstanbul* /bébek/ or as a *baby* /bebék/. The material in the pseudoword block was a pseudoword with both possible stress patterns /dedék/ and /dédek/. The stimuli were recorded in a semantically neutral frame sentence produced by a male native speaker of Turkish (from İstanbul, 30 years old) in an anechoic chamber, and were sampled at a rate of 44.1 kHz with 16 bits/per sample.

In order to keep the difference minimal across the blocks, the stimuli in the pseudoword block were created from the word block material by replacing the bilabial segment /b/ with dental segment /d/ in Praat (Boersma and Weenink, 2014)^3^. All stimuli were matched for duration (497 ms). In both word and pseudoword blocks, stimuli with iambic pattern always served as standards and stimuli with trochaic pattern as deviants. No manipulation was carried out for the standard; that is, the original realization of the iambic pattern was kept. The deviants (i.e., with trochaic pattern) were created out of the standards (i.e., with iambic pattern) by a cross-splicing technique. In order to get an equal ground for the comparison, it was important to ensure that the deviants were identical with the standard up to the onset of the second syllable; the first syllable /be/ in the word block and /de/ in the pseudoword block were therefore kept constant across standards and deviants. Then, the second syllable of the iambic pattern was spliced and acoustic manipulations were carried out. Since direction and amplitude of the acoustic change might influence the neural response, the second syllable was manipulated by lowering the acoustic parameters, which, in turn, lead to having iambic pattern always as standards and trochaic pattern as deviants. Given that word stress is about relations between syllables within words; that is prosodic features are characterized by a comparison of items in sequence (Lehiste, 1970), lowering the acoustic parameters of the second syllable in an iambic pattern should lead us to perceive it as a trochaic pattern.

Manipulation values were determined based on the proportions in the original trochaic pattern. By keeping the proportions between the first and second syllable in the trochaic pattern the same, the second vowel of the iambic pattern was manipulated by lowering (i) *f*_0_, (ii) spectral emphasis, (iii) duration, and (iv) all features, and four types of deviant stimuli were created. Manipulations did not lead to any lexical cost in the word block since both the standard and the deviants were real words. As the deviants maintained the same relations (proportions) as the original trochaic words, the manipulations yielded natural sounding words.

The *f*_0_-values were taken from pitch tracks and were resynthesized in Praat, which uses an acoustic periodicity detection algorithm based on an autocorrelation method (Boersma, 1993). *f*_0_ range settings were 75–600 Hz. Mean *f*_0_ was measured over each vowel. The vowel onset and offset were determined based on the pitch information and were free from the contextual influences of surrounding segments. Table 1 shows the *f*_0_ measurements in semitones relative to 1 Hz (st) in both stressed and unstressed positions.

**Table 1:** **Mean fundamental frequencies (*f*_0_) in semitones (st) and durations (in ms) of the vowels with final and non-final stress, and deviant-manipulation results**.

	**bebék (standard)**	**bébek (original)**	**bébek (deviant)**
/e/—σ1 mean *f*_0_ in st	85	89	85
/e/—σ2 mean *f*_0_ in st	87	83	79
Difference		6	6
/e/—σ1 Duration in ms	71	78	72
/e/—σ2 Duration in ms	72	63	57
Difference		15	15

**Figure 1:**
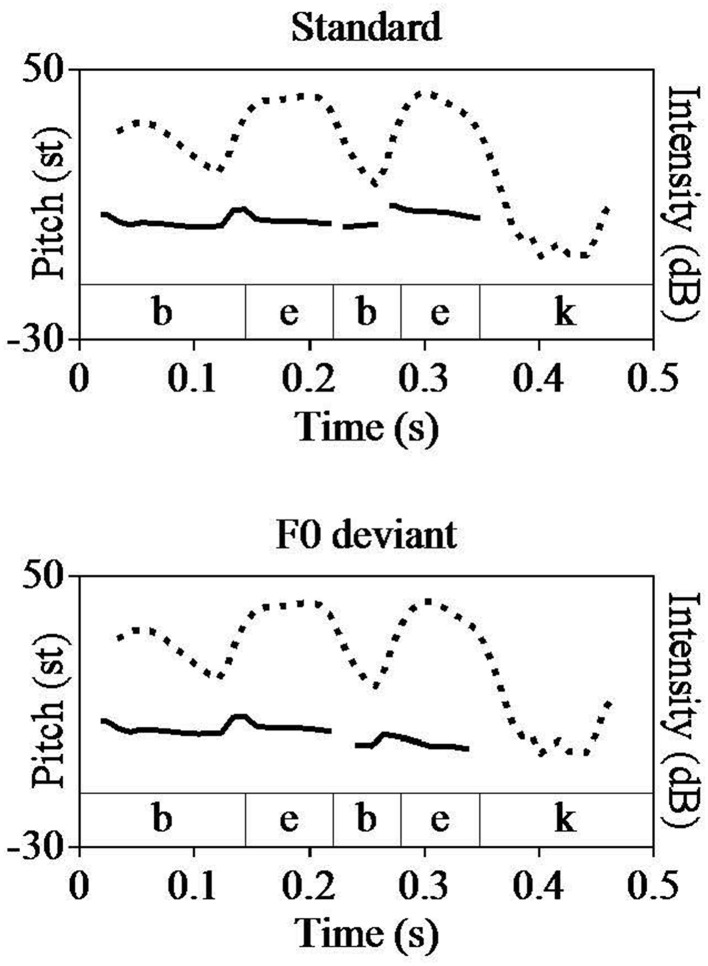
**Resynthesis of *f*_0_ in Praat**. Pitch (solid line) and intensity (dotted line) tracks of standard (top) and *f*_0_ deviant (bottom). The semitone scale (st; relative to 1 Hz) was used for pitch. F0, Fundamental frequency.

Since the first syllable was constant across the standards and deviants, *f*_0_ of the first vowel was 85 st in both stimuli (Table 1). The mean *f*_0_ difference between the stressed and unstressed vowels in the original word /*bébek*/ was 6 st (89 st-minus-83 st). In order to keep this proportion the same in the *f*_0_ deviant, *f*_0_ of the second vowel was set to 79 st (85 st-minus-6 st; Table 1). Figure 1 shows the pitch track of the resynthesized version in Praat.

The duration manipulation was performed in the same manner as the *f*_0_ manipulation. Vowel durations were measured in milliseconds (ms) and manipulated in Praat. Since the standard and deviants shared the same first syllable, duration of the first vowel is 72 ms in both stimuli (Table 1). The duration difference between the stressed and unstressed vowels in the original word /*bébek*/ is 15 ms (78 ms-minus-63 ms). In order to keep this proportion the same in the duration deviant, duration of the second vowel was set to 57 ms (72 ms-minus-15 ms; Table 1).

**Figure 2:**
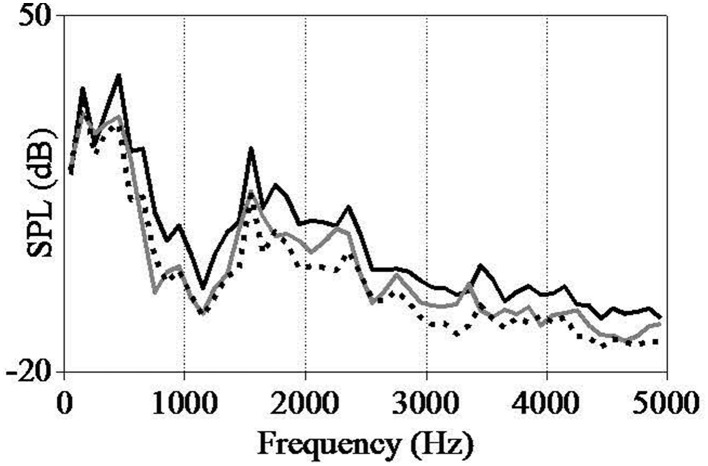
**Spectra of the second syllable in the standard (black line), the original word with non-final stress (gray line), and the spectral emphasis deviant (dotted line), using a 100 Hz bandwidth**.

The spectral emphasis manipulation was performed in Adobe Audition CS6. By comparing the second syllables of iambic and trochaic patterns, spectral emphasis differences were measured. By using the fast Fourier transform filter (fft-filter), the spectrum above the *f*_0_ was decreased 4 dB. The spectrum of each syllable is shown in Figure 2.

To create the fourth deviant, all features (*f*_0_, spectral emphasis, and duration) on the second vowel were manipulated at once.

### Procedure

The experiment was run using E-Prime (Psychology Software Tools, Pittsburgh, PA, USA). The stimuli were delivered via loudspeakers at a comfortable listening level of 60–65 dB at source. The stimuli were presented in a 5-stimulus auditory oddball paradigm (1 standard + 4 deviants). The frequently repeated standard stimulus (*p* = 8∕10) was randomly replaced by four rare deviant stimuli (*p* = 0.5/10 each), with at least two intervening standards between two consecutive deviants. Of 2000 stimuli, the number of standards was 1600 and the number of deviants was 400 (100 for each). The stimulus onset asynchrony was set at 1000 ms. A silent documentary was used to take the participants' attention off the auditory stimuli. The whole experiment lasted about 1 h 10 min.

### Electroencephalography recordings

The electroencephalography (EEG) signals were recorded at a sampling rate of 250 Hz, using NetStation 4.4 with a Net Amps 300 amplifier (Electrical Geodesic Inc., EGI, Eugene, Oregon, USA). The recordings were made from HydroCel Geodesic Sensor Net of 128 electrodes (EGI, Eugene, Oregon, USA) which employs a non-abrasion high-impedance application method. The impedance was kept below 50 kΩ at each electrode site as specified by EGI for this high impedance system. An online band-pass filter with cut-off frequencies at 0.1 and 70 Hz was applied. The CZ electrode was used as online reference and the ground reference had a centroparietal location.

### ERP data analysis

The EEG data was first filtered with a low-pass filter with a cutoff frequency of 30 Hz and with a high-pass filter with a cutoff frequency of 1 Hz. The channels were then re-referenced to both mastoids. The EEG data was segmented into epochs of 800 ms, time-locked to the onset of second syllable (200 ms before onset to 600 ms after onset). The onset of second syllable was used as the zero point in the data analysis because, given that the standards and deviants were same up to the second syllable, information about any difference between the standards and deviants would be present only after this point (see Pulvermüller et al., 2001; Pulvermüller and Shtyrov, 2003). A time window of 200 ms prior to the onset was used for baseline correction. Artifact rejection was set to remove activity exceeding ±100 μV at any channel. 60% of deviant trials had to be artifact-free in order for the retained material to form a valid base for the conclusions. A grand average was computed for each stimulus type for all participants and deviant-minus-standard subtraction signals were calculated for each deviant type.

### Statistical analysis

**Figure 3:**
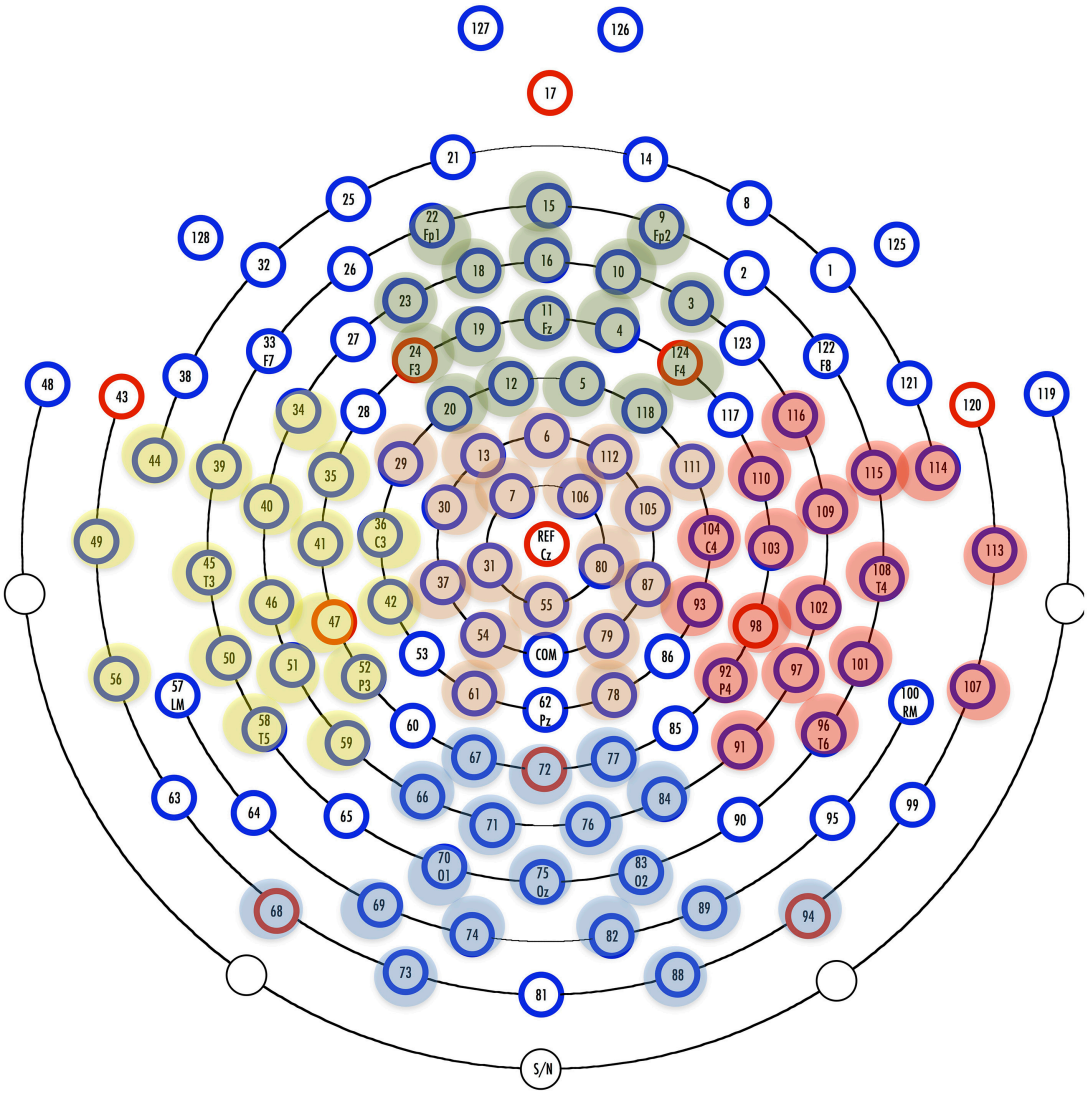
**128-channel HydroCel GSN and Regions of Interest (ROIs)**. Yellow marked electrodes, Left hemisphere; red marked electrodes, Right hemisphere; green marked electrodes, Frontal sites; orange marked electrodes, Central sites; Blue marked electrodes, Parietal sites.

Statistical analysis was performed in SPSS (International Business Machines Corp., Armonk, NY, USA). The electrodes were grouped together in five regions of interest (ROI): left, right, frontal, central, and parietal. The Figure 3 illustrates ROIs. The measurement window was determined by visual inspection of grand average difference waveforms. For MMN quantification, amplitudes were computed as a mean voltage within a 50-ms-window centered at the peak latency in the grand-average waveforms. Amplitude data extracted from deviant-standard subtraction curves was used for statistical analysis (see Näätänen and Winkler, 1999; Näätänen et al., 2007; Winkler, 2007; Kappenman and Luck, 2012).

A Three-way repeated-measures ANOVA with factors of Lexicality (two levels: word and pseudoword), Prosody (four levels: *f*_0_, spectral emphasis, duration, and all combined), and ROI (five levels: left, right, frontal, central, and parietal) was performed. If significant interactions occurred, follow-up ANOVAs were performed and the levels were then compared in *post-hoc* pairwise comparisons. SPSS Bonferroni adjusted *p*-values are reported for *post-hoc* comparisons. Additional two-tailed *t*-tests were used to look at topographical distributions closer. *P*-values are given with Greenhouse-Geisser correction in case of sphericity violations. Effect sizes are reported with η^2^ (partial η^2^).

## Results

### Event-related potential data

**Figure 4:**
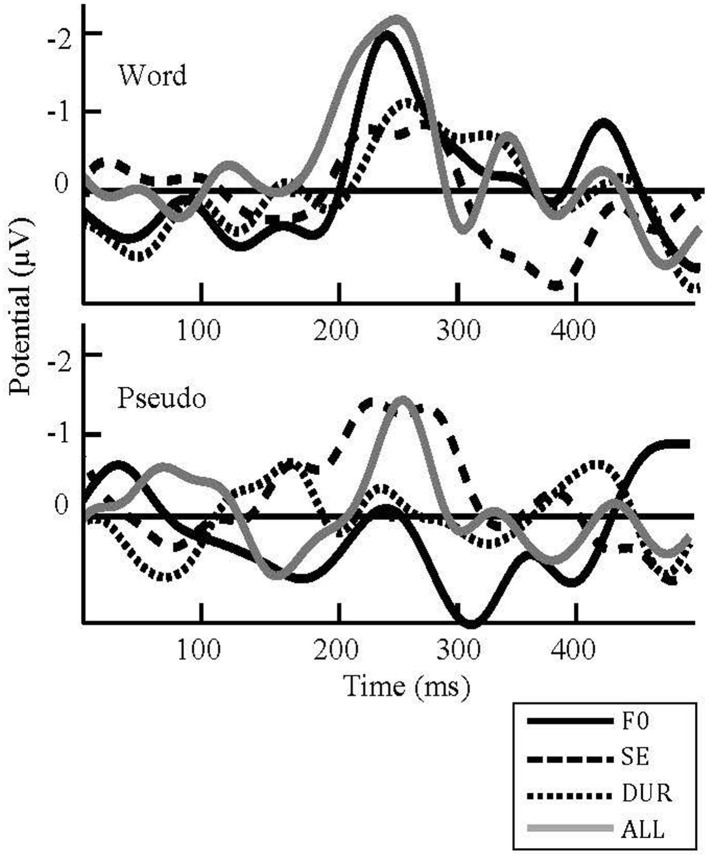
**Grand average difference waveforms for all deviants on FZ in word (top) and pseudoword (bottom) blocks**. F0, Fundamental frequency; SE, Spectral emphasis; DUR, Duration; ALL, Fundamental frequency, spectral emphasis and duration combined. Negativity is plotted upward.

**Figure 5:**
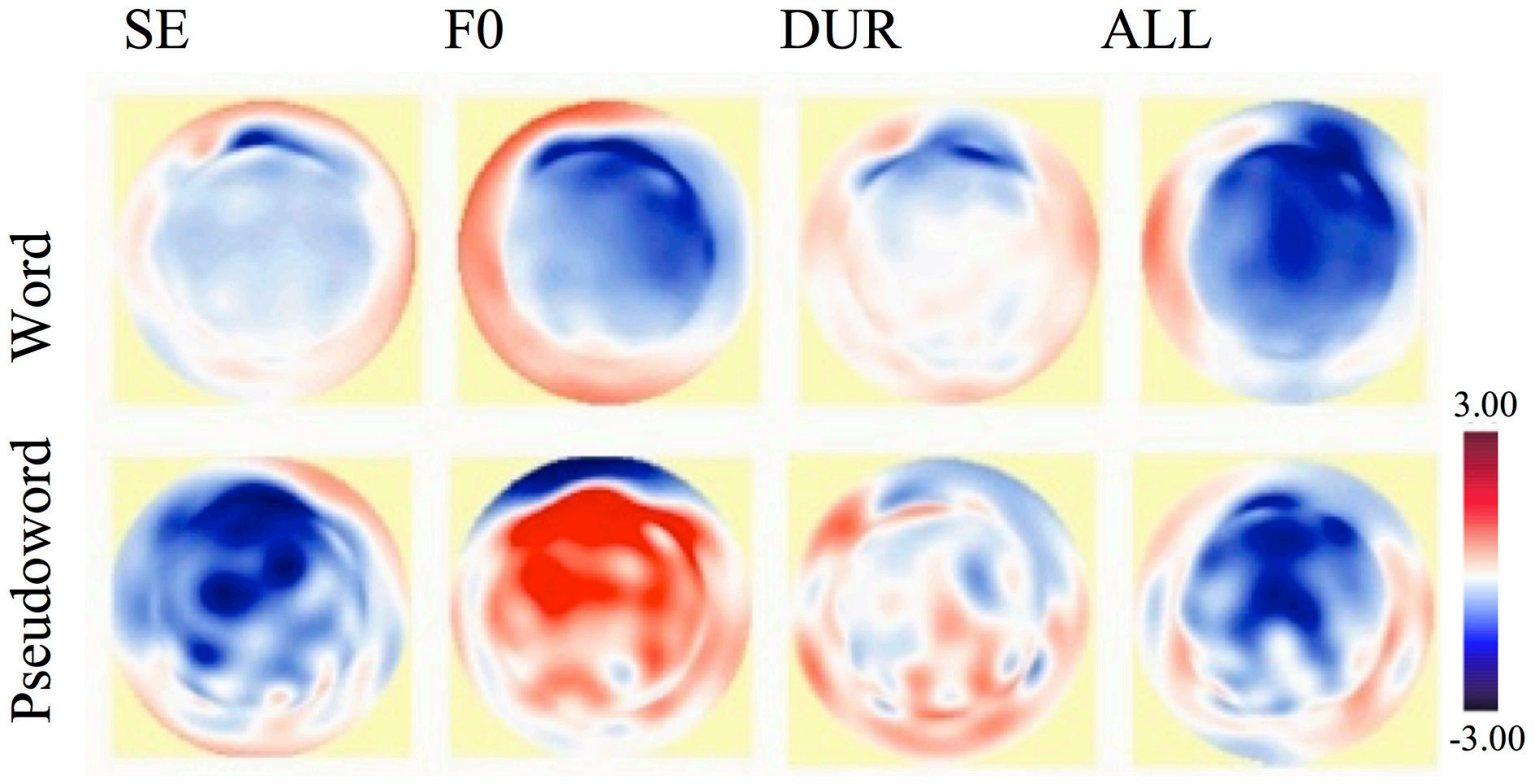
**Topographic difference maps at 240 ms after change onset for the word block (top) and for the pseudoword block (bottom): MMN for SE, for F0, for DUR, and for ALL, respectively, (from left to right)**. MMN, Mismatch negativity; F0, Fundamental frequency; SE, Spectral emphasis; DUR, Duration; ALL, Fundamental frequency, spectral emphasis and duration combined.

The grand average difference waves and scalp topographies for deviants are shown for word and pseudoword blocks in Figures 4, 5, respectively.

Changes in prosodic features elicited neural responses at around 230 ms after change onset (i.e., onset of second syllable) in both words (Figure 4, top) and pseudowords (Figure 4, bottom), confirming the brain's automatic response to any change in auditory sensory input. The processing differences between words and pseudowords were, on the other hand, reflected in the amplitude, polarity, and topography of the neural responses.

While eliciting a small negativity in words (Figure 5, top, left), the spectral emphasis deviant elicited a large negativity in pseudowords (Figure 5, bottom, left). These negativities were considered to be N200 responses. There are two negative components in the time range of N200: N2a or MMN and N2b (Näätänen and Gaillard, 1983; Näätänen, 1992). In contrast to the MMN, which reflects automatic processing and is elicited in unattended conditions, N2b is usually elicited in attended conditions (for a review, see Näätänen and Gaillard, 1983). However, when a stimulus deviation is wide, the N2b may also occur in unattended conditions (Näätänen et al., 1982). The presence of N2b has been indicated with a broad scalp distribution similar to that of auditory N100 (Näätänen, 1992). The spectral emphasis-related negativity in words was considered to be an MMN response whereas it was considered to be an N2b response in pseudowords due to its distribution in a wider area and due to the nature of the stimuli. This will be further discussed in Section Conclusion.

The most noticeable processing difference between words and pseudowords seems to be the *f*_0_-related activation; while eliciting a right-lateralized frontally-maximal negativity in words (Figure 5, top, second from left), the *f*_0_ manipulations elicited a frontal positivity in pseudowords. This *f*_0_-related positivity in pseudowords was considered to be a P300 response. If categorized according to their functional correlates and scalp distributions, P300 is typically divided into two subcomponents: P3a and P3b (Squires et al., 1975; Linden, 2005; Patel and Azzam, 2005; Polich, 2007). The P3a shows a frontally maximum scalp distribution and indexes the orienting of attention to unexpected events. The P3b shows a parietally maximum scalp distribution and indexes the updating of working memory. While the P3b is task relevant, the P3a is elicited without a task (Squires et al., 1975; Linden, 2005; Patel and Azzam, 2005; Polich, 2007). The positivity in pseudowords was argued to be a P3a response (Figure 5, bottom, second from left) due to task-independent elicitation and a frontal scalp distribution.

Although not so prominent, a duration-related frontally distributed negativity was present in both words (Figure 5, top, third from left) and pseudowords (Figure 5, bottom, third from left). The all-combined deviant elicited a fronto-centrally distributed negativity in both words (Figure 5, top, right) and pseudowords (Figure 5, bottom, right).

### Statistical data

**Figure 6:**
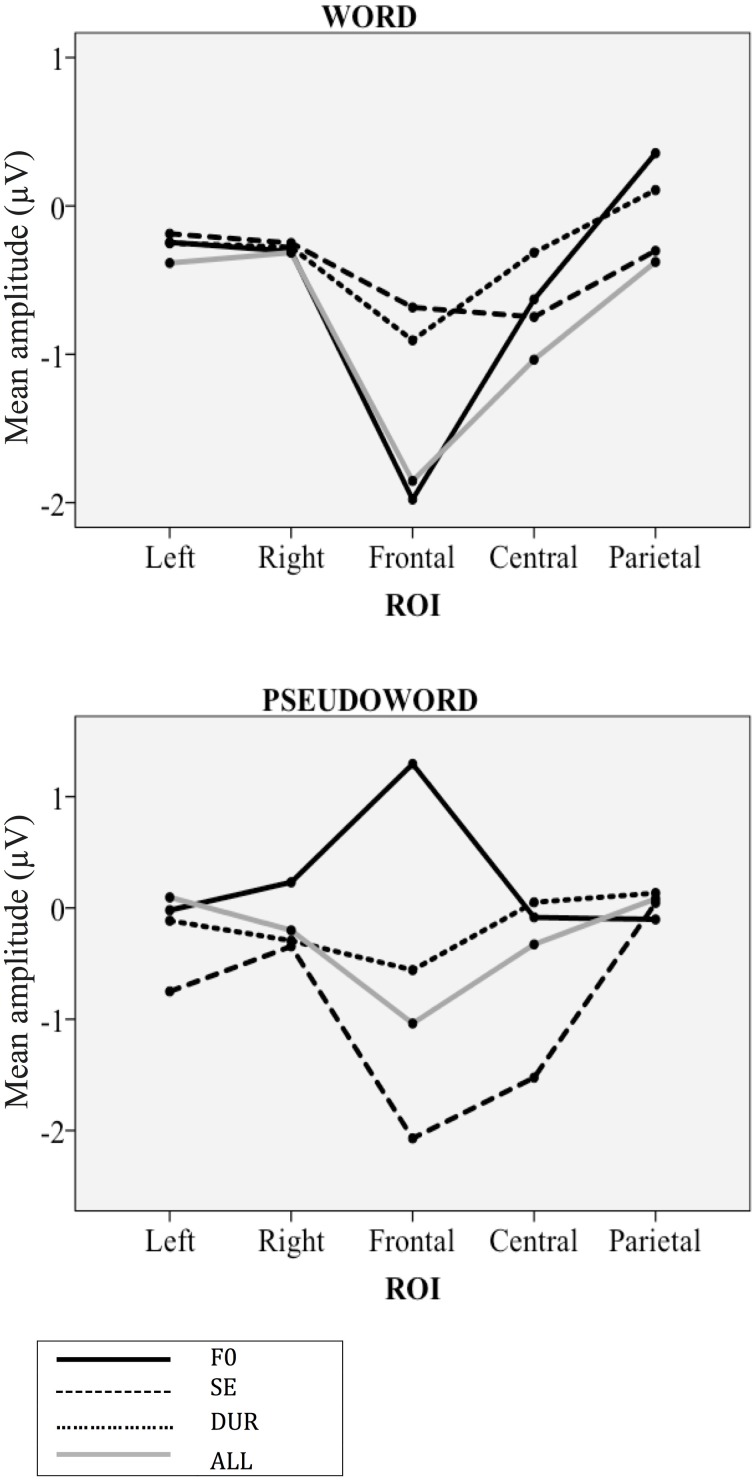
**Interactions in the word and pseudoword blocks, respectively**. ROI, Region of interest; F0, Fundamental frequency; SE, Spectral emphasis; DUR, Duration; ALL, Fundamental frequency, spectral emphasis and duration combined.

The statistical analysis in the time window 230–280 ms indicated a significant main effect of ROI [*F*_(4, 52)_ = 12.685, *p* < 0.001, η^2^ = 0.494]; a significant two-way interaction of Lexicality with Prosody [*F*_(3, 39)_ = 3.740, *p* = 0.019, η^2^ = 0.223], and a significant three-way interaction of ROI with Lexicality and Prosody [*F*_(12, 156)_ = 4.111, *p* < 0.001, η^2^ = 0.240; Table 2]. Figure 6 shows the interactions in words and pseudowords, respectively.

**Table 2:** **ANOVA and Bonferroni corrected *post-hoc* comparisons table for the time window 230–280 ms**.

**ANOVA**				**Pairwise**		
**Factor**	***F***	***p***	**η^2^**	**Main effect**	**ROI level**	***p***
ROI	*F*_(4, 52)_ = 12.685	0.000^*^	0.494	ROI	Left-Right	1.00
Lex	*F*_(1, 13)_ = 2.465	0.140	0.159		Left-Frontal	0.020^*^
Pro	*F*_(3, 39)_ = 2.233	0.100	0.147		Left-Central	0.008^*^
ROI × Lex	*F*_(4, 52)_ = 1.534	0.206	0.106		Left-Parietal	0.181
ROI × Pro	*F*_(12, 156)_ = 1.146	0.328	0.081		Right-Frontal	0.033^*^
Lex × Pro	*F*_(3, 39)_ = 3.740	0.019^*^	0.223		Right-Central	0.030^*^
ROI × Lex × Pro	*F*_(12, 156)_ = 4.111	0.000^*^	0.240		Right-Parietal	0.678
					Frontal-Central	0.599
					Frontal-Parietal	0.012^*^
					Central-Parietal	0.006^*^
**Follow-up 1**				**Lexicality**	**Prosody level**	***p***
Left: Lex × Pro	*F*_(3, 39)_ = 2.998	0.042^*^	0.187	Pseudo Frontal		
Right: Lex × Pro	*F*_(3, 39)_ = 2.115	0.114	0.140		*f*_0_-SE	0.010^*^
Frontal: Lex × Pro	*F*_(3, 39)_ = 4.484	0.008^*^	0.256		*f*_0_-DUR	0.146
Central: Lex × Pro	*F*_(3, 39)_ = 3.465	0.025^*^	0.210		*f*_0_-ALL	0.024^*^
Parietal: Lex × Pro	*F*_(3, 39)_ = 1.727	0.177	0.117		SE-DUR	0.592
					SE-ALL	0.359
					DUR-ALL	1.00
**Follow-up 2**				Central	*f*_0_-SE	0.110
Left-Word: Pro	*F*_(3, 39)_ = 0.358	0.784	0.027		*f*_0_-DUR	1.00
Left-Pseudo: Pro	*F*_(3, 39)_ = 2.729	0.085	0.173		*f*_0_-ALL	1.00
Frontal-Word: Pro	*F*_(3, 39)_ = 0.783	0.511	0.057		SE-DUR	0.042^*^
Frontal-Pseudo: Pro	*F*_(3, 39)_ = 7.688	0.000^*^	0.372		SE-ALL	0.113
Central-Word: Pro	*F*_(3, 39)_ = 1.047	0.382	0.075		DUR-ALL	1.00
Central-Pseudo: Pro	*F*_(3, 39)_ = 6.339	0.001^*^	0.328			

*ROI, Region of Interest; Lex, Lexicality; Pro, Prosody; f_0_, Fundamental frequency; SE, Spectral emphasis; DUR, Duration; ALL, Fundamental frequency, spectral emphasis and duration combined*.

* *p < 0.05*.

The Bonferroni-corrected *post-hoc* pairwise comparisons for the main effect of ROI revealed that the MMN over frontal sites (*M* = −0.974 μV, *SD* = 0.209) was larger than the MMN over the left hemisphere (*M* = −0.232 μV, *SD* = 0.069, *p* = 0.020), over the right hemisphere (*M* = −0.220 μV, *SD* = 0.069, *p* = 0.033) and over parietal sites (*M* = −0.007 μV, *SD* = 0.113, *p* = 0.012); the MMN over central sites (*M* = −0.577 μV, *SD* = 0.137) was larger than the MMN over the left hemisphere (*p* = 0.008), over the right hemisphere (*p* = 0.030) and over parietal sites (*p* = 0.006; Table 2). The ROI effect showed that the distribution of MMN was largest over frontal and central sites.

The Follow-up 1 analysis confirmed the interaction between Lexicality and Prosody in the left hemisphere [*F*_(3, 39)_ = 2.998, *p* = 0.042, η^2^ = 0.187], at frontal sites [*F*_(3, 39)_ = 4.484, *p* = 0.008, η^2^ = 0.256], and at central sites [*F*_(3, 39)_ = 3.465, *p* = 0.025, η^2^ = 0.210; Table 2]. Prosody reached significance in pseudowords only at frontal [*F*_(3, 39)_ = 7.688, *p* < 0.001, η^2^ = 0.372] and central sites [*F*_(3, 39)_ = 6.339, *p* = 0.001, η^2^ = 0.328]. The Bonferroni-corrected *post-hoc* pairwise comparisons for the Follow-up 2 analysis at frontal sites indicated significant differences between *f*_0_ (*M* = 1.294 μV, *SD* = 0.504) and spectral emphasis (*M* = −2.071 μV, *SD* = 0.554, *p* = 0.010), between *f*_0_ and all-combined (*M* = −1.038 μV, *SD* = 0.549, *p* = 0.024). The Bonferroni-corrected *post-hoc* pairwise comparisons for the Follow-up 2 analysis at central sites indicated significant difference between spectral emphasis (*M* = −1.524 μV, *SD* = 0.309) and duration (*M* = 0.051 μV, *SD* = 0.295, *p* = 0.042; Table 2). Pairwise comparisons in the pseudoword block showed that only *f*_0_ elicited positivity at frontal sites; that duration did not elicit any negativity at central sites, and that the negativity for spectral emphasis was still prominent at central sites.

Additional two-tailed *t*-tests were used to compare the amplitudes obtained for deviants in words against those in pseudowords in each ROI (Table 3). In the right hemisphere, *f*_0_ elicited negativity in words (*M* = −0.306 μV, *SD* = 0.150) while eliciting positivity in pseudowords (*M* = 0.231 μV, *SD* = 0.225, *p* = 0.011). In frontal sites, *f*_0_ elicited negativity in words (*M* = −1.978 μV, *SD* = 0.813) while eliciting positivity in pseudowords (*M* = 1.294 μV, *SD* = 0.504, *p* = 0.001). In central sites, spectral emphasis elicited larger negativity in pseudowords (*M* = −1.524 μV, *SD* = 0.309) than in words (*M* = −0.748 μV, *SD* = 0.231, *p* = 0.044). *f*_0_-related MMN activation was right-lateralized in words. *f*_0_-related difference between words and pseudowords was maximal over frontal sites. The spectral emphasis-related activation was more pronounced over central sites in pseudowords in comparison to words.

**Table 3:** **Two-tailed *t*-tests with the amplitudes obtained for deviants in words and pseudo words in each ROI**.

**Prosody**	**Lexicality level**	***p***
**LEFT**		
*f*_0_	Word-pseudo	0.244
SE	Word-pseudo	0.055
DUR	Word-pseudo	0.592
ALL	Word-pseudo	0.052
**RIGHT**		
*f*_0_	Word-pseudo	0.011^*^
SE	Word-pseudo	0.717
DUR	Word-pseudo	0.916
ALL	Word-pseudo	0.591
**FRONTAL**		
*f*_0_	Word-pseudo	0.001^*^
SE	Word-pseudo	0.098
DUR	Word-pseudo	0.739
ALL	Word-pseudo	0.512
**CENTRAL**		
*f*_0_	Word-pseudo	0.074
SE	Word-pseudo	0.044^*^
DUR	Word-pseudo	0.351
ALL	Word-pseudo	0.085
**PARIETAL**		
*f*_0_	Word-pseudo	0.056
SE	Word-pseudo	0.207
DUR	Word-pseudo	0.928
ALL	Word-pseudo	0.226

*f_0_, Fundamental frequency; SE, Spectral emphasis; DUR, Duration; ALL, Fundamental frequency, spectral emphasis and duration combined*.

* *p < 0.05*.

## Discussion

By recording neural responses to changes in fundamental frequency, spectral emphasis and duration, the present study investigated for the first time to what extent each perceptual correlate of stress is utilized for lexical access in Turkish. It was predicted that the neural responses would indicate differences in the contributions of fundamental frequency, spectral emphasis and duration in stress perception and lexical access. Given that studies on Turkish stress have so far investigated only the phonetic correlates in acoustic studies (Konrot, 1981; Levi, 2005; Pycha, 2006), this study makes an important contribution to the literature by investigating the perceptual correlates with an electrophysiological approach. In contrast to the previous acoustic studies, which employed complex words and therefore failed to avoid morphological effects, the present study investigated the perceptual correlates of Turkish stress using simplex (monomorphemic) words. It is also worth noting that the present study used a spectral emphasis measure to better assess the importance of loudness considering that the contribution of higher frequency bands to the perceived intensity is much greater (Sluijter and van Heuven, 1996; Sluijter et al., 1997; Heldner, 2003). It is therefore the first study to introduce spectral emphasis measures to Turkish word stress and to an ERP study.

The findings indicated that the MMN responses were maximal over frontal and central scalp locations in line with the previous findings (Näätänen and Winkler, 1999; Näätänen et al., 2007). The findings further indicated that changes in prosodic features elicited neural responses at around 230 ms after change onset in both words and pseudowords, confirming the brain's automatic response to any change in auditory sensory input which typically peaks at 150–250 ms from change onset (Näätänen et al., 2007).

However, there were processing differences of prosodic information between words and pseudowords. First, while eliciting a frontal MMN in words, spectral emphasis manipulations elicited a fronto-centrally maximal N2b in pseudowords. The presence of N2b in pseudowords was indicated by its topography, which usually shows a broad scalp distribution somewhat similar to that of an auditory N100 (Näätänen, 1992). Apart from the topographical factor, another factor favoring the N2b-effect interpretation was that the negativity had a larger amplitude in pseudowords: If it were an MMN, reflecting an acoustic processing, then the amplitude of negativity would be the same in words and pseudowords since they are identical in their acoustic features; if it were an MMN, reflecting lexical processing, then the amplitude of negativity would be larger in words than in pseudowords. However, negativity was larger in the amplitude of pseudowords. Given that the presence of N2b has been suggested to be a result of stimulus-directed attention (Näätänen et al., 2007), negativity to spectral emphasis manipulations in pseudowords might be simply due to a larger attentional load, indicating that the spectral emphasis change was rather unexpected in the absence of lexical processing. The spectral emphasis might, therefore, play a crucial role in lexical processing. Given that this study is the first to introduce spectral emphasis measures into ERP, previous studies cannot explain the current results; the role of spectral emphasis in lexical processing is therefore a subject for further ERP research.

Second, while eliciting a frontally maximal MMN in words, fundamental frequency manipulations elicited a frontal positivity in pseudowords. This fundamental frequency-related positivity in pseudowords was considered to be a P3a response since P3a shows a task-independent, frontally maximum activation (Squires et al., 1975; Linden, 2005; Patel and Azzam, 2005; Polich, 2007). Alternatively, one could argue positivity to be a P200 response as a reflection of pitch sensitivity. The peak latency of P200 is at about 200 ms and the scalp distribution is typically noted to be maximal over central regions (Näätänen, 1990). However, the positivity here is later in latency and shows a frontal distribution. Moreover, the positivity elicited here is believed to be not just a reflection of pitch change but rather an attention orientation. It is therefore argued to be a P3a response. Given that P3a reflects involuntary allocation of attention to salient changes (Squires et al., 1975; Näätänen, 1990; Linden, 2005; Polich, 2007), this fundamental frequency-related P3a in pseudowords could be due to attention orientation to pitch change. In contrast to the MMN that indexes lexical as well as acoustic processing, P3a activation is limited to physical parameters and reflects only the acoustic processing. The fact that fundamental frequency changes elicited an MMN in words whereas eliciting a P3a in pseudowords suggests therefore that fundamental frequency is lexically specified in Turkish.

Although not as prominent, duration-related frontally distributed MMN was present in both words and pseudowords. Duration is therefore claimed to be present as an acoustic cue for word stress perception although it does not make a significant contribution to automatic lexical access. This is not surprising given that the vowel length is not phonemically distinctive in Turkish (Kornfilt, 1997; Nimz, 2015). Previous acoustic studies (Konrot, 1981; Levi, 2005; Pycha, 2006) ruled out duration as an acoustic correlate in Turkish word stress. In this regard, this study makes an important contribution to the literature by indicating duration as a perceptual correlate. However, although being a potential perceptual correlate, the role of duration in automatic lexical access could not be established.

The deviant combining fundamental frequency, spectral emphasis, and duration elicited negativity in both words and pseudowords. Combined with previous findings, this suggests that it is individual prosodic features that differentiate lexical processing from non-lexical processing rather than combined features. This, further, provides answer to the question whether prosodic features are processed separately or holistically in the auditory system. A number of MMN studies indicated that acoustic features are represented separately, indicating independent storage of these features (for a review see Caclin et al., 2006). Separate processing of acoustic features has been favored by studies of MMN generation location (Giard et al., 1995; Rosburg, 2003). This argument was further supported by studies that showed additivity of responses to single deviants; that is, the MMN elicited by multiple deviants can be predicted by the sum of the MMNs of the corresponding single deviants (Wolff and Schröger, 2001). However, deviants with three deviating features might not follow the MMN additivity argument and might elicit a smaller MMN than predicted by the sum of the single deviants, indicating complex processing of simultaneously deviating features (Paavilainen et al., 2001). MMN amplitude is argued to index the probability of a specific feature rather than the probability of feature combinations (Deacon et al., 1998). In line with these findings, present results support a model where acoustic features are processed separately.

The brain does not only detect prosodic changes but also uses them in lexicality decisions; there is a difference in how the brain treats prosodic cues depending on lexicality. Prosodic changes consistently elicit MMN in words while eliciting different components in pseudowords depending on the deviating cue. If the MMN responses were to indicate pre-lexical processing only, then the neural responses would be the same in words and pseudowords since they are identical in their acoustic features. Different components in pseudowords reflect acoustic-change effects on the neural responses rather than language-related effects. Prosodic feature manipulations, in the absence of lexical processing, trigger an increase in attentional load and cause reevaluation of what was heard.

In summary, the present study is the first to demonstrate the neural correlates of prosodic features in Turkish word stress and their contribution to lexical access. The findings indicate that there are memory traces for prosodic information in the brain and that they play a significant role in lexical access without any given context. The findings show that segmentally identical Turkish words can indeed be distinguished on the sole basis of prosodic features. In line with the previous acoustic studies, which found fundamental frequency as being the most salient cue in marking stressed syllables (Konrot, 1981; Levi, 2005; Pycha, 2006), fundamental frequency has been found as the most prominent perceptual correlate. This finding is potentially in agreement with the phonetic criterion, which claims Turkish to be a pitch-accent language rather than a stress-accent language (Underhill, 1986; Levi, 2005). Apart from confirming the perceptual salience of fundamental frequency, the present study also indicates the contribution of fundamental frequency in lexical access and therefore argues that fundamental frequency is lexically specified in Turkish. It should again be noted that the role of spectral emphasis in lexical access is subject to further research. In short, fundamental frequency is argued to be the most prominent perceptual correlate and lexically specified due to the fact that it elicited the largest negativity in words, and that the most remarkable processing difference between words and pseudowords was related to fundamental frequency.

## Conclusion

Perceptual correlates of Turkish word stress and their contribution to lexical access were studied. Neural responses to changes in fundamental frequency, spectral emphasis and duration were recorded in Turkish words and pseudowords. The findings indicate that memory traces for Turkish words are indeed activated on the sole basis of prosodic information. The presence of prosodic representations in lexical representations has a differential effect on the processing of prosodic changes. The manipulations of prosodic features, in the absence of lexical processing, increase the attentional load and cause reevaluation of the auditory stimuli. Fundamental frequency as the most salient perceptual correlate contributes to lexical access and is therefore lexically specified in Turkish.

## Author contributions

HZ, MH, IS: The conception and design of the work; the analysis of the data; drafting the work and revisiting it critically; final approval of the version to be published.

### Conflict of interest statement

The authors declare that the research was conducted in the absence of any commercial or financial relationships that could be construed as a potential conflict of interest.

## Footnotes

^1^According to the phonetic criteria proposed in Beckman (1986), pitch-accent languages employ only fundamental frequency when marking the stressed syllable, while stress-accent languages may also use intensity and duration.

^2^Capital letters indicate that segment is unspecified and might vary according to vowel harmony.

^3^http://www.praat.org
